# Bionanocomposite of Dual Antioxidant and Protease Function by Co‐Immobilization of Tannic Acid and Papain on Anionic Clays

**DOI:** 10.1002/chem.202500846

**Published:** 2025-04-30

**Authors:** Zsuzsanna D. Konya, Adel Szerlauth, Istvan Szilagyi

**Affiliations:** ^1^ MTA‐SZTE Lendület Biocolloids Research Group Department of Physical Chemistry and Materials Science Interdisciplinary Excellence Centre University of Szeged Szeged H‐6720 Hungary

**Keywords:** antioxidants, co‐immobilization, papain, protease, tannic acid

## Abstract

The synchronized operation of antioxidants and enzymes is necessary for the proper functioning of living organisms and various industrial processes. The drawback that these biomolecules are susceptible to several environmental factors can be overcome by their immobilization on appropriate host materials. Here, molecular antioxidant tannic acid (TA) and papain enzyme (PPN) were co‐immobilized on layered double hydroxide (LDH) nanoparticles to enhance their stability and facilitate their combined function. In the sequential adsorption method governed by electrostatic interactions, TA was first adsorbed on oppositely charged LDH (LDH/TA) and then, PPN was immobilized on the surface of the nanoparticles (LDH/TA/PPN). The optimal TA and PPN doses were determined by systematic size and charge measurements of the particles in dispersions to obtain stable colloids in each preparation step. The co‐immobilization of the biomolecules was confirmed by spectroscopy methods. The resulting LDH/TA/PPN nanocomposite exhibited remarkable antioxidant and protease activities. The dual biological function together with the considerable colloidal stability make the LDH/TA/PPN material a promising candidate in various processes in academia and more applied disciplines, where simultaneous antioxidant and proteolytic functions are desired.

## Graphical abstract

Development of highly stable bionanocomposite dispersions of antioxidant and protease activities was achieved by co‐immobilization of TA and papain on LDH nanoparticles.

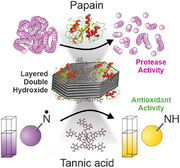

## 1 Introduction

The simultaneous application of antioxidants and enzymes is essential in both physiological and industrial processes, as the former ones prevent or interrupt the harmful effects of reactive oxygen species (ROS),^[^ ^1^ ^]^ while the latter ones accelerate chemical reactions through their specific biocatalytic functions.^[^ ^2^ ^]^ ROS‐induced oxidative stress is linked to the development of numerous diseases including inflammation, cancer, and neurodegenerative conditions.^[^ ^3^ ^]^ Antioxidants inhibit oxidative stress by balancing free radicals, but if ROS concentration becomes significant, lipid peroxidation, protein, and DNA damage can occur.^[^ ^4^ ^]^ Lifestyle issues and increasing environmental pollution are associated with elevated free radical production,^[^ ^5^ ^]^ which cannot be compensated by the defense system of endogenous antioxidants and thus, the support from exogenous sources is becoming increasingly important to maintain adequate antioxidant concentrations.^[^ ^6^ ^]^ The exogenous antioxidants include vitamins, flavonoids, and other polyphenolic systems, while the most important endogenous ones are enzymes.^[^ ^1b^ ^]^ Along with their biomedical applications,^[^ ^7^ ^]^ antioxidants are frequently used in various industrial sectors, e.g., in the food industry as additives and preservatives^[^ ^8^ ^]^ as well as in dermatology and cosmetic products, as active ingredients to prevent the rapid oxidative degradation of these products.^[^ ^9^ ^]^ However, the low water solubility, high reactivity, degradability, and susceptibility to environmental effects may limit the industrial application of both exogenous and endogenous antioxidants.^[^ ^10^ ^]^

Proteases—hydrolase enzymes capable to break covalent bonds of biomolecules—account for nearly 60% of the enzymes market.^[^ ^2^, ^11^ ^]^ They directly affect countless biological processes including DNA replication and transcription, cell differentiation, wound healing, blood clotting, inflammation, and fertility by controlling interactions between proteins, regulating signaling processes and forming new bioactive molecules.^[^ ^12^ ^]^ Disruption of their balance leads to the development of cancerous, neurodegenerative, cardiovascular, and inflammatory diseases.^[^ ^13^ ^]^ Beside their physiological role, proteases are among the most valuable commercially available enzymes, as they are used in detergent,^[^ ^14^ ^]^ pharmaceutical,^[^ ^15^ ^]^ food,^[^ ^2b^ ^]^ and cosmetic^[^ ^16^ ^]^ products, but they also play an important role as biocatalysts in syntheses of fine chemicals.^[^ ^11^, ^13^ ^]^ However, similar to antioxidant enzymes, the harsh temperature, pressure, and pH conditions applied in industrial processes may cause denaturation and thus, the loss of catalytic activity of proteases.^[^ ^17^ ^]^ Due to the rapid development in biotechnology, it is now possible to produce and modify most enzymes cost‐effectively, but, because of the considerable decrease in activity, selective separation from the reaction mixture and reusability are still important challenges in the field.^[^ ^11^, ^18^ ^]^

The physical or chemical attachment of biomolecules including enzymes on solid supports has been a frequently used method to eliminate the unfavorable features mentioned above.^[^ ^18^, ^19^ ^]^ In general, such an immobilization can be classified by the type of carrier (e.g., natural,^[^ ^20^ ^]^ organic,^[^ ^21^ ^]^ or inorganic^[^ ^22^ ^]^ materials) and by the forces involved, e.g., covalent, electrostatic, or van der Waals interactions were reported.^[^ ^18^ ^]^ Layered double hydroxides (LDHs) are widely applied inorganic host materials, known for their brucite‐like layered crystal structure consisting of various divalent and trivalent metal cations in certain ratios, which are surrounded by hydroxyl groups in octahedral geometry.^[^ ^23^ ^]^ The positive net charge caused by the trivalent cations is compensated by exchangeable hydrated interlayer anions.^[^ ^24^ ^]^ The large specific surface area of LDHs allows molecules to be bound on the surface of the particles,^[^ ^25^ ^]^ but their layered structure also facilitates the intercalation within the lamellae.^[^ ^26^ ^]^ Their biocompatibility, cost‐effective synthesis, high mechanical, and chemical stability make them favorable host materials in biomedical and industrial applications.^[^ ^24^, ^26^, ^27^ ^]^ LDH‐based immobilized systems possess the advantages of heterogeneous catalysts, such as simple separation from the reaction mixture,^[^ ^18^, ^19^ ^]^ and long‐term retained activity,^[^ ^10^, ^11^ ^]^ compared to homogeneous counterparts.

Tannic acid (TA) and papain (PPN) are both used in dermatology and the cosmetic industry due to their anti‐inflammatory, wound healing, and antimicrobial effects.^[^ ^7^, ^28^ ^]^ Due to the polyphenolic structure, TA is an efficient antioxidant of remarkable radical scavenging activity to reduce oxidative stress. For instance, addition of TA into cosmetic products gave rise to improved anti‐aging and anti‐wrinkle properties,^[^ ^29^ ^]^ while poly‐TA containing materials are reported as sustainable hydrophobic coating agents with remarkable biodegradability.^[^ ^30^ ^]^ Beside, PPN, as a protease enzyme, can be an excellent exfoliating agent in various creams and facemasks.^[^ ^16^ ^]^ Due to their joint antibacterial, antiviral, and antioxidant nature, TA and PPN are promising co‐additives in biodegradable packaging materials.^[^ ^31^ ^]^ Since LDHs are also used to stabilize biofilms,^[^ ^27a^ ^]^ combining the three (TA‐PPN and LDH) substances in the same composite is a well‐seeming research direction to develop multifunctional biocatalytic materials for various industrial and biomedical use. Although, previous reports on immobilization of TA^[^ ^25^, ^32^ ^]^ or PPN^[^ ^33^ ^]^ on LDH particles individually are available, but no literature data exist on their co‐immobilization.

**Scheme 1:**
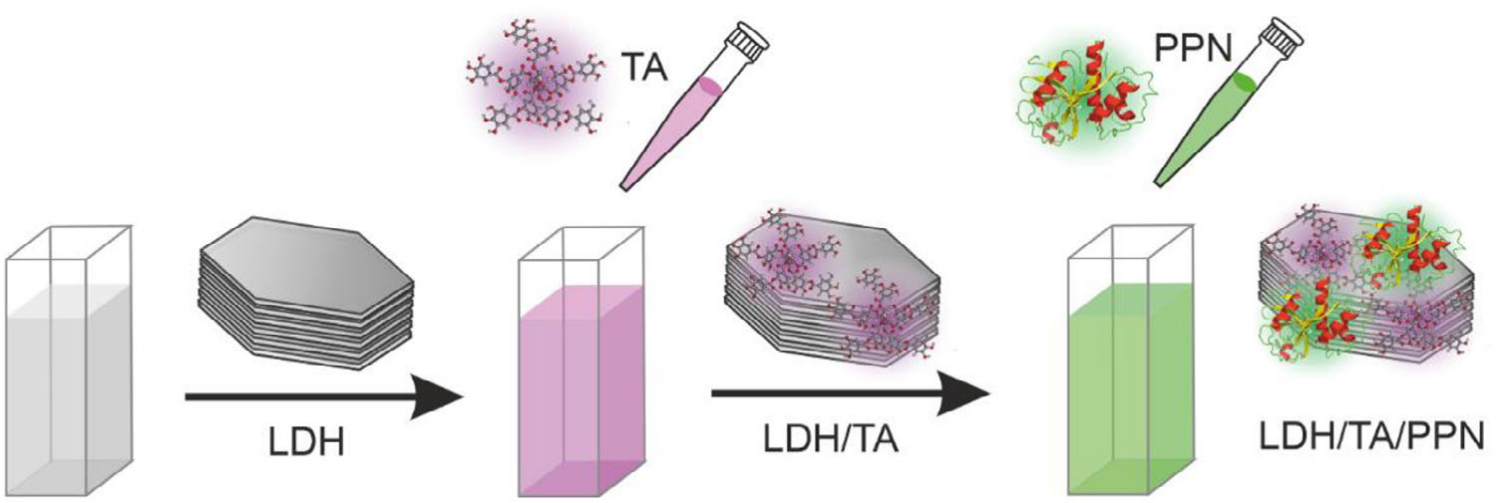
Illustration of the preparation route of the LDH/TA/PPN hybrid nanomaterial.

To tackle with the above‐mentioned challenges, a nanocomposite consisting of TA, PPN, and LDH was developed in the present work (Scheme 1). TA and PPN were chosen because the main goal was to achieve dual antioxidant and protease function by co‐immobilization on a single nanoparticle support to suggest future application in food manufacturing processes, where radical scavenge and protein cleavage are both desired.^[^ ^2^, ^34^ ^]^ The synthetic procedure was carried out with the sequential adsorption method, in which the colloidal properties of the nanoparticle systems were optimized in each step. The composition and structure of the composite formed was confirmed, while the radical scavenging and hydrolytic activities were assessed.

## 2 Results and Discussion

### 2.1 Co‐immobilization of TA and PPN on LDH

**Figure 1:**
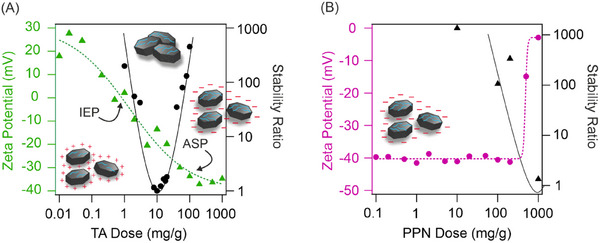
Change in zeta potential (Equation 1) and stability ratio (Equation 3) of LDH particles as a function of the TA (A) and PPN (B) dose. The experiments were performed at 10 mg/L LDH concentration and 1 mM NaCl background electrolyte. The mg/g unit refers to mg TA or PPN per one gram of particle. The lines are eye guides. Note that stability ratio of one indicates rapid particle aggregation controlled solely by the diffusion of the particles.

TA and PPN were immobilized on LDH nanoparticles via the sequential adsorption method based on electrostatic interactions between the particles and the biomolecules. First, the optimal TA dose was determined for the preparation of the LDH/TA compound in aqueous dispersions aiming high colloidal stability. Particle charge was characterized by zeta potentials (Equation 1) and size with hydrodynamic radius (Equation 2) determined with electrophoretic and dynamic light scattering, respectively. In these measurements, TA doses were systematically changed and the LDH concentration was kept constant. The colloidal stability was expressed in terms of stability ratio (Equation 3), which are shown together with the potential data for the LDH/TA system in Figure 1A.

At low TA doses, the zeta potential of about +30 mV is due to the positive structural charge of the LDH. With increasing TA content, the potentials decreased and charge reversal occurred at appropriately high concentrations. The TA dose, at which the adsorbed molecules neutralize the particle charge (so‐called isoelectric point, IEP) was observed at around 1 mg/g, while the adsorption saturation plateau (ASP) starts at 100 mg/g. The latter value indicates the maximum TA amount that can be immobilized on the particles. These results clearly indicate the high affinity of TA to the LDH surface. Similar charge reversal of LDH was observed with ellagic acid molecular antioxidant earlier.^[^ ^10a^ ^]^

Similar to other particles undergone charge reversal in dispersions,^[^ ^21^, ^35^ ^]^ the DLVO (Derjaguin, Landau, Verwey, and Overbeek) theory provided a qualitative explanation for the tendency in the stability ratio versus TA dose data as follows. At low antioxidant concentrations, the electrostatic repulsion between the highly charged particles stabilized the dispersions, i.e., the hydrodynamic radii remained constant (Figure S1A in the Supporting information and stability ratio was not measurable. By increasing the TA content, onset of aggregation was observed (i.e., stability ratios were measured) at the IEP, while it was the fastest, i.e., stability ratio was around unity, around 10 mg/g. In this regime, the hydrodynamic radii increased rapidly with time (Figure S1A). A quantitative deviation from the DLVO prediction is that the fastest aggregation should take place at the IEP, while it was observed at an order of magnitude higher dose. This phenomenon clearly indicates the presence of non‐DLVO forces.^[^ ^36^ ^]^ Nevertheless, we do not have direct experimental evidence to reveal the origin of such interactions. By further increasing the TA dose, the dispersion became stable at 100 mg/g (at the onset of ASP), at which dose hydrodynamic radii remained again constant (Figure S1A), aggregation did not occur. Based on the above dispersion characterization, 200 mg/g TA dose was applied in further studies (denoted as LDH/TA later).

In the next step, PPN was immobilized on the surface of the LDH/TA nanoparticles in a similar approach (Figure 1B). Unlike in the case of TA, the zeta potentials were constant within the experimental error up to a high PPN dose of 200 mg/g. It is likely that the constant potential data is due to the low charge of the PPN enzyme (IEP 8.8^[^ ^37^ ^]^), under the conditions studied and the high charge of the LDH/TA particles. Hence, PPN adsorption does not change the zeta potentials significantly in the lower dose regime. Also note that zeta potentials reflect the charging behavior at the slip plane and not directly at the surface.^[^ ^38^ ^]^ The particle charge started to increase above this dose, however, charge reversal could not be observed. The trend in the stability ratios was in good agreement with the zeta potential results considering the DLVO model. Accordingly, at low PPN doses, the hydrodynamic radii did not change (Figure S1B) indicating stable dispersions, while above 200 mg/g, in line with the decreasing magnitude of the surface charge, the dispersion became unstable and faster particle aggregation was indicated by the decrease in the stability ratios. Based on the results of the above charge and size assessments, 200 mg/g PPN dose was selected. Therefore, the LDH/TA/PPN used for further studies contained 200 mg/g TA and 200 mg/g PPN. This composite is negatively charged and possess remarkable colloidal stability.

### 2.2 Composition and Structure of LDH/TA/PPN

The adsorbed amount of TA and PPN was measured indirectly by the Bradford assay.^[^ ^39^ ^]^ This chemical test reaction is used for the determination of protein concentration in solution. Accordingly, Coomassie Brilliant Blue dye forms a blue complex with the free protein molecules (e.g., PPN), which reaction can be detected by a spectrophotometer. The absorption maximum of the dye is initially at 465 nm, which disappears with increasing PPN concentration, while the new absorption maximum of the dye‐PPN complex appears at 595 nm. This method can also be used to determine the concentration of TA, as complex formation also occurs with tannins.^[^ ^40^ ^]^ In the experiments, the LDH/TA and LDH/TA/PPN dispersions were centrifuged to eliminate the particles and the TA or PPN content in the supernatant was measured. In this way, the adsorbed amount can be calculated knowing the total analytical concentrations of the biomolecules.

The absorbance spectra of the TA‐dye complex were recorded in the range of 0–100 mg/L TA concentration (Figure S2A). The new band, related to the formation of dye‐TA complex, appeared at 660 nm. The change in the absorbance values was linear in the 0–10 mg/L concentration range, while above this level the curve turned into saturation (Figure S2B). The free TA concentration in the supernatant measured was less than 5% of the total amount of TA added to the sample. This result confirmed that most of the TA molecules adsorbed on the LDH surface forming LDH/TA and only negligible amount of antioxidants remained in the bulk solution during immobilization.

**Figure 2:**
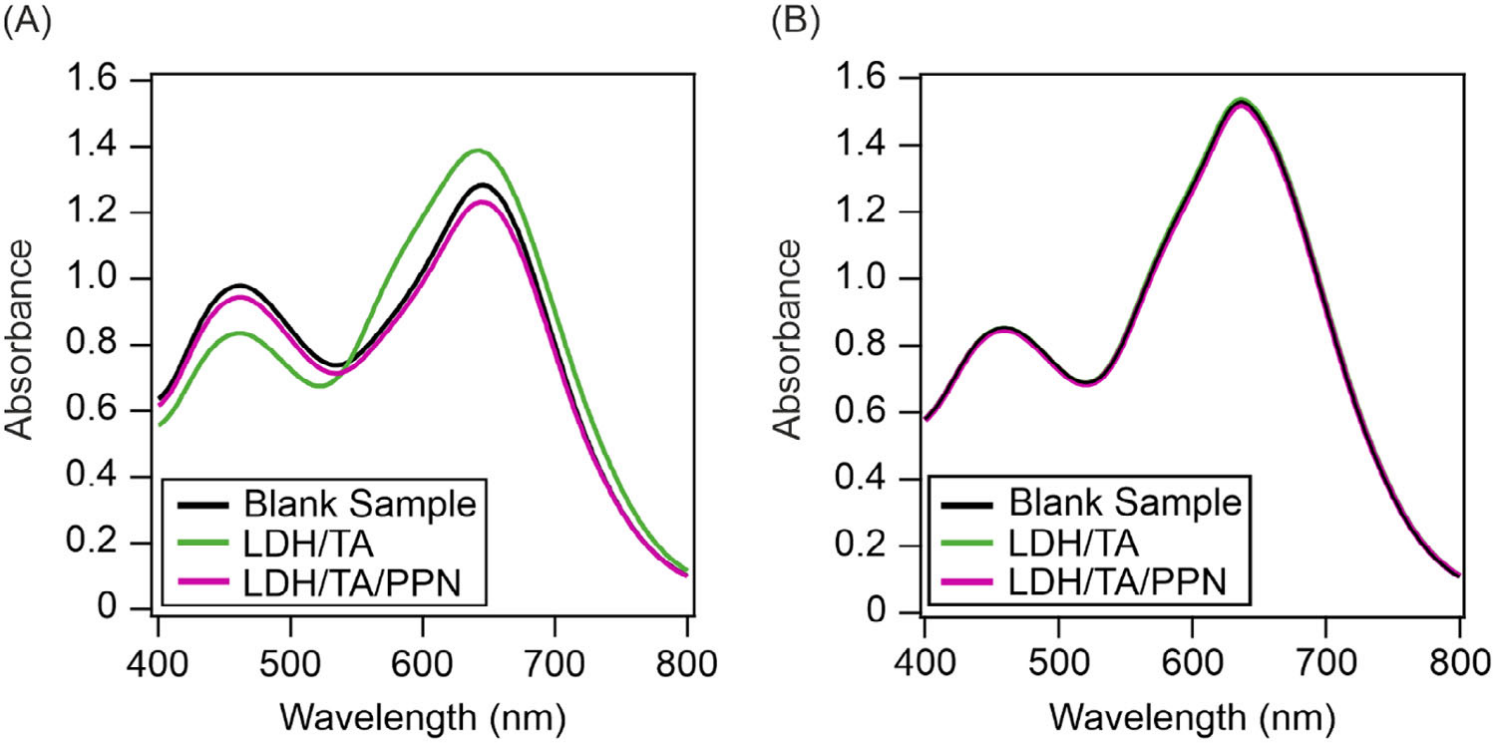
Spectra of the supernatant of LDH/TA and LDH/TA/PPN compared to solutions containing neither TA nor PPN (blank sample) measured with (A) freshly prepared (25 °C) and (B) heat treated (2 hours, 50 °C) systems.

Similar results were obtained for the PPN systems (Figure S2C). Calibration curve was established from the absorbance values measured for the newly appeared band at 595 nm and the change in absorbance values was linear in the 0–20 mg/L concentration range (Figure S2D). Experiments performed with the supernatant of the LDH/TA/PPN dispersion did not yield positive result, i.e., free PPN could not be determined (Figure 2A). These findings clearly confirm that the adsorption of TA and PPN was quantitative in the LDH/TA and LDPH/TA/PPN systems and the excess amount of the biomolecules was negligible in the dispersion.

For the temperature‐dependent investigations, the above tests were repeated with LDH/TA and LDH/TA/PPN samples incubated at 50 °C for 2 hours. The spectra of heat‐treated samples did not differ from the ones recorded with the blank solution (without added TA or PPN), which indicated that no desorption occurred during the heat treatment of the nanocomposites (Figure 2B).

**Figure 3:**
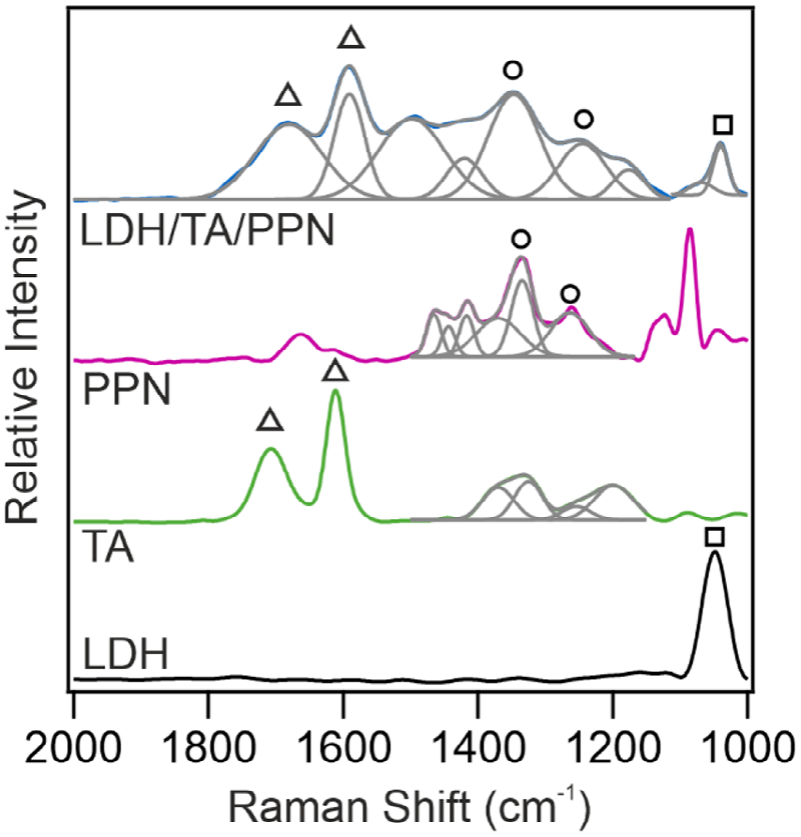
Raman spectra of LDH, TA, PPN, and LDH/TA/PPN recoded in dried stage. The peak deconvolutions are also shown. The symbols indicate characteristic vibration bands discussed in the text.

Raman spectroscopy measurements (Figure 3) further confirmed the immobilization of the biomolecules. The characteristic peaks of both TA and PPN appeared on the spectrum of the LDH/TA/PPN nanocomposite. Accordingly, in the LDH and LDH/TA/PPN samples, bands appeared at 1048 cm^−1^ and 1039 cm^−1^ (□), which were assigned to the stretching mode vibration of N─O in interlamellar nitrate ions.^[^ ^25^ ^]^ The peak in the TA spectrum at 1612 cm^−1^ corresponds to the stretching mode vibration of C═C bond in the aromatic ring, while the band at 1707 cm^−1^ was assigned to the stretching mode vibration of the carbonyl (C═O) group in the ester bond (△). For LDH/TA/PPN, a slight shift was observed in the position of these bands (from 1612 cm^−1^ to 1591 cm^−1^ and from 1707 cm^−1^ to 1680 cm^−1^), which can be attributed to the immobilization of TA and thus, the hindered vibration of its functional groups. Beside the above‐mentioned data, the spectrum of TA also possessed bands at 1324 and 1370 cm^−1^ wavenumbers assigned to the bending mode vibrations of the phenolic hydroxyl groups, and peaks at 1197 and 1255 cm^−1^ attributed to the stretching mode vibration of C─C and C─O bonds.^[^ ^41^ ^]^ The Raman bands observed at 1244 and 1345 cm^−1^ in the LDH/TA/PPN spectrum were ascribed to the Amide III vibrations of PPN (°). Similar to TA, upon immobilization, significant shift in the band positions could be observed for LDH/TA/PPN, which indicates the successful adsorption of PPN on the LDH/TA surface. Other characteristic peaks observed in the spectrum of PPN at 1663 cm^−1^ was assigned to the Amide I vibrations, while the peaks in the 1369–1466 cm^−1^ region correspond to the stretching mode vibration of C─H bonds. The observed Raman bands and their assignments are collected in Table S1 (see Supporting Information). Besides, the spectra of LDH and LDH/TA/PPN materials show two bands at 470 cm^−1^ and 550 cm^−1^ wavenumbers (Figure S3) related to the symmetric stretching vibrations of Mg─O and Al─O in the LDH structure.^[^ ^42^ ^]^ The fact that the Raman spectrum of LDH/TA/PPN contains all the characteristic bands of LDH confirms that no change in the microstructure of LDH occurred during preparation of LDH/TA/PPN.

The above light scattering and spectroscopy studies on the LDH/TA/PPN and its subsystems unambiguously confirm the successful co‐immobilization of TA and PPN in the hybrid material. The data indicates that the major interactions between the components are of electrostatic origin, but other forces, such as hydrogen bonding and hydrophobic effects may be also present.

### 2.3 Radical Scavenging Activity

**Figure 4:**
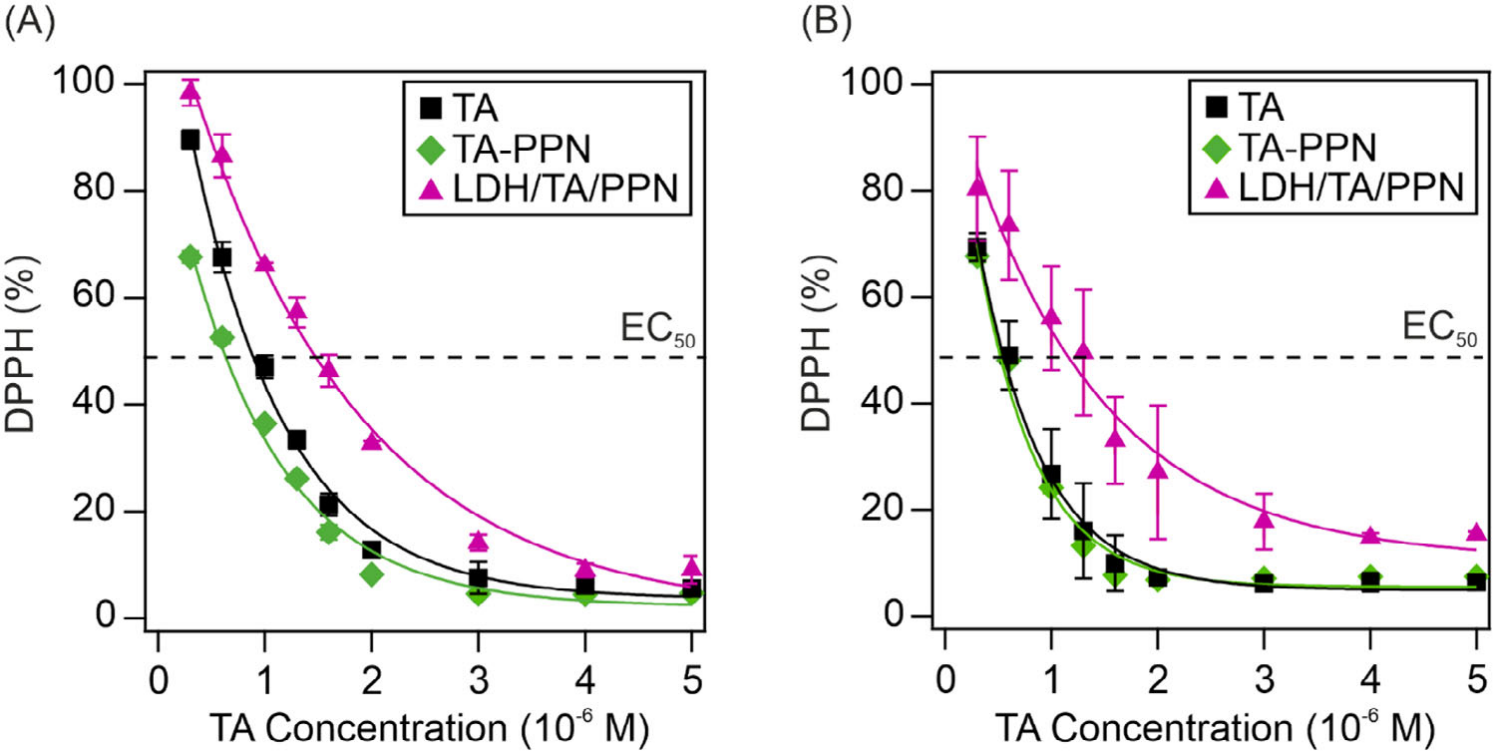
Radical scavenging activity of TA, TA‐PPN mixture (no LDH added), and LDH/TA/PPN nanocomposite at (A) 25 °C and (B) 50 °C. The lines are mathematical fits used to determine the EC_50_ values.

The antioxidant activity of TA, TA‐PPN mixture (without LDH), and LDH/TA/PPN particles was determined using the 2,2‐diphenyl‐1‐picrylhydrazyl (DPPH) assay.^[^ ^43^ ^]^ During the measurements, the stable DPPH radical is reduced by the antioxidant molecules, which resulted in a color change in the solution. The change in absorbance values can be monitored at the absorption maximum of DPPH at 517 nm wavelength. The tests were carried out at two different temperatures, namely at 25 and 50 °C. To compare the performances of the substances studied, the DPPH (%), i.e., the remaining DPPH in solution (Equation 4), was plotted against the added TA concentration (Figure 4).

With increasing TA dose, the DPPH (%) gradually decreased and a very similar trend was observed in all three (TA, TA‐PPN, and LDH/TA/PPN) systems. Despite immobilization, TA retained its radical scavenging properties and the samples possessed significant antioxidant activity in all cases. For LDH/TA/PPN, the slight decrease in activity may be due to the interaction of DPPH with the particle surface through a possible steric inhibiting effect, as hydroxyl groups and aromatic rings surrounding the nitrogen atom may interact with the surface functionalities. Although, a slight difference can be observed between the remaining DPPH (%) data for the TA and TA‐PPN samples, the deviation is not significant, as the EC_50_ values (antioxidant concentration needed for the decomposition of 50% of the DPPH radicals, shown in Table S2) were very similar.

The samples showed similar activity within experimental error after incubation at 50 °C (Figure 4B) indicating that the heat treatment had no discernible impact on the radical scavenging activity of the systems, which was further confirmed by the calculated EC_50_ values shown in Table S2. Note, that LDH and PPN displayed no antioxidant activity at either temperature in the concentration range applied, therefore, the radical scavenging activity of the composite is attributed to the TA molecules in each case.

### 2.4 Proteolytic Activity

**Figure 5:**
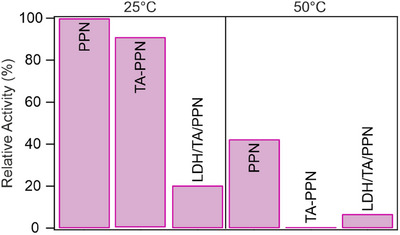
Relative protease activity values of PPN, TA‐PPN mixture (no added LDH), and LDH/TA/PPN nanocomposite at 25 °C (left side data) and 50 °C (right side data) normalized to the activity of PPN determined at 25 °C temperature. Note that TA and LDH showed no protease activity; therefore, the hydrolytic function of the materials shown is attributed to the PPN enzyme.

To assess the peptide bond breaking ability of the nanocomposite, a nonspecific protease assay based on the Lowry method^[^ ^44^ ^]^ was performed on the PPN, TA‐PPN mixture, and LDH/TA/PPN systems. In the tests, casein acts as a substrate, which is hydrolyzed by the protease in solution. The released tyrosine amino acid, as the product of the hydrolysis, reacts with the added Folin‐Ciocalteu reagent leading to color change that can be monitored by recording the absorbance values at 660 nm (Figure S4). From the absorbance values, the amount of released tyrosine can be calculated, which is then used to determine the protease activity (Equation 5) of the substance in question. After incubation at 50 °C, the activity of PPN decreased by a factor of two indicating that part of the enzymes denaturated and lost their protease activity due to higher temperature (Figure 5).

At 25 °C temperature, the PPN and TA‐PPN samples did not show significant difference in protease function. Nevertheless, in contrast to PPN, the TA‐PPN mixture essentially lost its activity after incubation at 50 °C. The TA is expected to react with the enzymes under this condition, since it is able to form complexes with the majority of proteins. However, research on the impact of complexation on enzyme activity frequently yields conflicting results. In earlier reports,^[^ ^45^ ^]^ it was demonstrated that complex formation boosts enzyme activity at low TA concentrations, whereas activity decreases at higher TA levels. Besides, elevated TA doses results in agglomeration of the molecules, which can alter the structure of enzymes and block their active sites. In this instance, heat treatment resulted in a notable decrease in activity of the TA‐PPN mixture. A similar phenomenon was observed in the case of TA‐lysozyme complexes, which gradually lost their activities with increasing temperature.^[^ ^45b^ ^]^ However, there is a lack of systematic investigations on the formation and behavior of TA‐PPN complexes or on the impact of complexation on protease activity. Accordingly, it can only be inferred that potential interaction between TA and PPN results in the loss of protease activity of PPN at higher temperatures.

The LDH/TA/PPN nanocomposite showed lower protease activity compared to the free enzyme in solution, in line with previous results in other systems obtained by immobilization of PPN.^[^ ^11^, ^21^, ^46^ ^]^ The reasons for the decreased activity include the adsorption of enzyme molecules in inadequate orientation on the surface, the formation of PPN multilayers^[^ ^21^ ^]^ and the presence of additional steric inhibiting factors preventing the substrate‐catalyst interactions. The activity decreased further after incubation at 50 °C, which may result from the denaturation of part of the PPN macromolecules due to heat treatment, similar to the individual enzyme. However, it is important to note that LDH/TA/PPN still exhibits considerable (significantly higher than the TA‐PPN mixture) activity at 50 °C. This fact indicates that immobilization improved the protease activity of PPN compared to the free counterpart in bulk solution. Since the results of the Bradford assay discussed earlier confirmed that the composition of the LDH/TA/PPN nanomaterial did not change upon heat treatment, the formation of TA‐PPN complexes can presumably be controlled or prevented by the co‐immobilization of these molecules on a single particle leading to retained catalytic activity in protein hydrolysis.

## 3 Conclusions

In summary, TA and PPN were successfully immobilized on the surface of LDH nanoparticles by the sequential adsorption method to obtain the LDH/TA/PPN nanocomposite of high colloidal stability and multienzymatic function. Systematic charge and size measurements in dispersions allowed the determination of the optimal dose of TA and PPN during immobilization. The attachment of the biomolecules was confirmed by an appropriate colorimetric assay and the results revealed that the adsorption was quantitative and no partitioning took place between the bulk solution and the particle surface. The presence of TA and PPN in the nanocomposite was further proven by spectroscopy by assigning the characteristic vibrational and rotational peaks of TA and PPN to the ones appeared in the LDH/TA/PPN spectrum. The TA retained its radical scavenging ability after immobilization and the nanocomposite possessed high antioxidant activity even at elevated temperature. The PPN lost some of its activity during the adsorption and heat treatment due to structural changes upon interaction with the particle surface and heat induced denaturation, respectively. Overall, a dual active nanocomposite of excellent colloidal stability was developed. The developed LDH/TA/PPN nanocomposite possesses both hydrolase and antioxidant functions, i.e., it is able to cleave proteins and scavenge radicals, making it a promising candidate in industrial biocatalysis, where the above discussed dual function is a significant advantage. Further investigations with this hybrid material may include in vitro and in vivo assessment of oxidative stress decreasing ability.

## 4 Experimental Section

### Materials

Magnesium nitrate hexahydrate (Mg(NO_3_)_2_×6 H_2_O), aluminum nitrate nonahydrate (Al(NO_3_)_3_×9 H_2_O), sodium hydroxide solution (4 M), TA, ethanol absolute (99,8%), DPPH, casein acc. to Hammarsten, L(‐)‐tyrosine, trichloroacetic acid (TCA), anhydrous sodium carbonate (Na_2_CO_3_), dipotassium phosphate trihydrate (K_2_HPO_4_×3 H_2_O), sodium acetate trihydrate (CH_3_COONa×3 H_2_O), anhydrous calcium acetate (Ca(CH_3_COO)_2_), Coomassie Brilliant Blue G‐250 dye, and orthophosphoric acid (85%) were purchased from VWR in analytical grade. Papain from Carica Papaya (PPN) was purchased from Genfi Cucco and Folin‐Ciocalteu reagent (2N) was purchased from Sigma Aldrich in analytical grade. Ultrapure water was produced by an Adrona B30 purification system.

### Preparation of LDH Nanoparticles

Single‐phase LDH particles of 2:1 Mg(II)‐to‐Al(III) ratio were synthetized using the coprecipitation method.^[^ ^24^ ^]^ Magnesium nitrate (0.2 M) and aluminium nitrate (0.1 M) mixed salt solution was vigorously stirred under N_2_ atmosphere. The pH was set to 10 by adding the appropriate amount of 4 M NaOH solution. After a 1‐hour‐long stirring, the slurry was centrifuged (4200 rpm, 10 minutes) and washed with deionized water three times. The resulting solid was redispersed and transferred into an autoclave for an overnight heat‐treatment at 120 °C. The final product was centrifuged (4200 rpm, 10 minutes) and washed with deionized water three times followed by drying at 50 °C in a ventilated oven overnight. The successful formation of the LDH nanoparticles was confirmed with powder X‐ray diffractometry (PXRD). The characteristic diffractions, shown in Figure S5 in the Supporting Information, indicated that highly crystalline and single‐phase lamellar Mg/Al LDH was produced.^[^ ^25^ ^]^

### Light Scattering

A Litesizer 500 (Anton Paar) instrument was used for electrophoretic and dynamic light scattering measurements to determine zeta potential and hydrodynamic radius (R_h_) data, respectively. The instrument is equipped with a laser (685 nm wavelength) operating at 40 mW power. The measurements were performed at 25 °C in backscattering mode (175° scattering angle). The Ω‐shaped cuvettes (Anton Paar) were used for the zeta potential measurements, while disposable plastic cuvettes (VWR International) for hydrodynamic size determination.

For electrophoretic measurements, samples containing 10 mg/L LDH were mixed with the appropriate amounts of TA and PPN, which ranged between 0–1000 mg/g doses. Prior to measurements, the samples were allowed to rest overnight to achieve complete adsorption on the LDH surface. The samples contained 1 mM NaCl as background electrolyte. Zeta potentials (ζ) were calculated using the Smoluchowski equation:^[^ ^38^ ^]^

(1) $$\mu =\frac{\varepsilon \varepsilon_{0}\xi }{\eta }$$

where μ is the electrophoretic mobility, ε is the relative permittivity of the solvent, ε_0_ is the permittivity of vacuum, and η is the dynamic viscosity of the solvent. The reported potential values were the average of 3 measurements with an average error of ±5 mV.

The hydrodynamic radius (*R*_h_) of the particles was determined by dynamic light scattering immediately after mixing the above components. Otherwise, the same sample preparation protocol was used as in the electrophoretic measurements. The R_h_ values were obtained with the Einstein–Stokes equation from the diffusion coefficient (D):^[^ ^47^ ^]^

(2) $$R_{h}=\frac{k_{B}T}{6\Pi \eta D}$$

where *k*_B_ is the Boltzmann constant and *T* is the absolute temperature. Stability ratio (W) values were calculated from time‐resolved dynamic light scattering measurements data with an established protocol for particle aggregation experiments using the following formula:^[^ ^10^, ^21^, ^22^, ^35^, ^46^ ^]^

(3) $$W\;=\frac{k_{\mathrm{fast}}}{k}\;$$

where *k*_fast_ is the fast aggregation regime determined at 1 M NaCl concentration, at which repulsive electrostatic forces are screened and every collision causes aggregate formation, while k is the actual aggregation rate. The *R*_h_ values were determined with ±5 nm precision and the error of the stability ratio data measurements is 10%.

### Determination of TA and PPN Concentration

The amount of TA and PPN in solution was determined by the Bradford assay, based on the absorption shift of the Coomassie Brilliant Blue dye that binds to several groups of biomolecules including proteins^[^ ^39^ ^]^ and hydrolysable tannins.^[^ ^40^ ^]^ In brief, 10 mg of the dye was dissolved in a mixture containing 5 mL of 95% ethanol and 10 mL of 85% phosphoric acid. Then the samples were completed to 100 mL with ultrapure water. Stock solutions of TA and PPN of 500 mg/L concentration were prepared to establish the calibration curves. A 1.6 mL aliquot of the dye was added to increasing amounts of TA and PPN solutions and the reaction mixture was then completed with ultrapure water to a final volume of 2 mL. The visible spectra of the samples were recorded after 5 minutes in the 400─800 nm wavelength range using a Genesys 10S spectrophotometer. For the composites, LDH/TA and LDH/TA/PPN solutions containing 1000 mg/L of LDH and 200 mg/L of TA and PPN were prepared and centrifuged for 5 minutes at 5000 rpm. Thereafter, 0.4 mL of the supernatant was added to 1.6 mL of the dye solution and thus, the TA and/or PPN concentration in the reaction mixture was 40 mg/L. The visible spectra were recorded after 5 minutes equilibration time. Note that the error of the above protocol is about 5─10% depending on the systems under study.

### Radical Scavenging Activity Assessment

To determine the free radical scavenging activity of the composite, the DPPH assay^[^ ^43^ ^]^ was used for TA, TA‐PPN mixture, and LDH/TA/PPN systems. A DPPH solution with a concentration of 48 mg/L was prepared by dissolving DPPH powder in 95% ethanol. TA, TA‐PPN mixture, and LDH/TA/PPN dispersion containing 500 mg/L of LDH and/or 100 mg/L of TA and/or PPN were prepared. To achieve full adsorption of the biomolecules on the LDH surface, the dispersions were prepared a day prior to the radical scavenging measurements. In a typical sample preparation, 1.5 mL of the DPPH solution was mixed with 1.5 mL of solutions or dispersions containing the required concentration of the biomolecules (0‐5 µM). The absorbance values were read at the maximum absorbance of the DPPH molecule at 517 nm wavelength after 30 minutes. The remaining DPPH amount in the solutions (*DPPH* %) was calculated with the following formula:

(4) $$DPPH\;\left(\%\right)=\frac{A}{A_{0}}100$$

where *A* is the final absorbance value after 30 minutes reaction time, and *A*_0_ is the absorbance of the starting DPPH solution.

### Protease Activity Measurements

To determine the protease activity of the nanocomposite, a protocol based on the Lowry method was used.^[^ ^44^ ^]^ The assay uses casein as a substrate, which is hydrolyzed by the protease content of the solution. The hydrolysis products include tyrosine, which forms a blue solution when Folin‐Ciocalteu reagent is added. The calibration curve was obtained by mixing different amounts of 1.1 mM tyrosine standard and 2 M Folin‐Ciocalteu reagent. The tyrosine concentration of these solutions ranged between 6–70 µM. The samples were then incubated for 30 minutes at 37 °C and centrifuged for 10 minutes at 10,000 rpm. The absorbance of the supernatants was recorded at 660 nm. The assay was performed on PPN, TA‐PPN mixture, and LDH/TA/PPN nanocomposites. In all measurements, the stock solutions contained 250 mg/L PPN, TA, and LDH. The PPN solution and LDH dispersion were prepared in 50 mM and pH = 7.5 phosphate buffer, while TA solution was prepared using 10 mM and pH = 7.5 acetate buffer (5 mM Na‐acetate and 5 mM Ca‐acetate). The reaction mixture contained 0.5 mL casein solution (0.65 w/v% in phosphate buffer) and different amounts (0‐0.1 mL) of PPN. After 20 minutes equilibration, the reaction was terminated by adding 0.5 mL of 110 mM TCA solution. The final volume of the samples was completed to 1.1 mL with ultrapure water followed by incubation for 30minutes at 37 °C and centrifugation for 10 minutes at 10,000 rpm. Thereafter, 0.75 mL of 500 mM Na_2_CO_3_ and 0.1 mL of 2 M Folin‐Ciocalteu reagent were added to 0.3 mL of the supernatant, and after another incubation period of 30 minutes at 37 °C, the samples were centrifuged for 10 minutes at 10,000 rpm. The absorbance values of the samples were measured at 660 nm. Protease activity was calculated from the absorbance values recorded at this wavelength. The amount of tyrosine formed in the hydrolysis of casein was calculated with the calibration curve and thus, the protease activity was determined. The protease activity of the systems was expressed as follows:

(5) $$\mathrm{Protease}\;\mathrm{activity}\;\left(\frac{U}{\mathrm{mL}}\right)=\frac{n_{\mathrm{tyr}}V_{\mathrm{reaction}}}{t_{\mathrm{reaction}}V_{\mathrm{PPN}}V_{660\mathrm{nm}}}$$

where *n*_tyr_ is the amount of released tyrosine determined with the calibration plot, *V*_reaction_ is the final volume of the reaction mixture (1.15 mL), *t*_reaction_ is the time of the reaction (20 minutes), *V*_PPN_ is the volume of PPN containing solution and *V*_660_ _nm_ is the volume of samples used for the colorimetric detection (1 mL). One unit of protease activity corresponds to the amount of PPN needed for the formation of 1 µg/mL tyrosine per minute. For temperature dependent measurements, the assays described above were repeated with samples incubated at 50 °C for 2 hours. Before performing the measurements, the samples were left to cool down to room temperature. The accuracy of the above protocol is within 15%.

### Structural Characterization Methods

The PXRD measurement was conducted with a Philips type powder diffractometer operating with CuKα radiation (0.1542 nm wavelength) in the 2θ range of 5–80°. The Raman spectra were recorded with a Bruker Senterra II Raman microscope equipped with a 50 mW light source of 785 nm wavelength and the average of 32 spectra, collected with an exposition time of 4 seconds, was reported.

## Conflict of Interests

The authors declare no conflict of interest.

## Supporting information



Supporting Information

## Data Availability

The data that support the findings of this study are available from the corresponding author upon reasonable request.
