# Supplementary material for: Bionanocomposite of Dual Antioxidant and Protease Function by Co‐Immobilization of Tannic Acid and Papain on Anionic Clays
Source: Chemistry. 2025 Apr 30;31(31):e202500846. doi: 10.1002/chem.202500846 (PMC12133637; doi:10.1002/chem.202500846)
Supplement: Supplementary file 1 — Supporting Information [file CHEM-31-e202500846-s001.pdf]

Supporting Information (SI)

Bionanocomposite of Dual Antioxidant and Protease Function by Co-immobilization of Tannic Acid and Papain on Anionic Clays

Zsuzsanna D. Konya, Adel Szerlauth, Istvan Szilagyi*

MTA-SZTE Lendület Biocolloids Research Group, Department of Physical Chemistry and Materials Science, Interdisciplinary Excellence Centre, University of Szeged, H-6720 Szeged, Hungary

**Corresponding author. Email: szistvan@chem.u-szeged.hu*

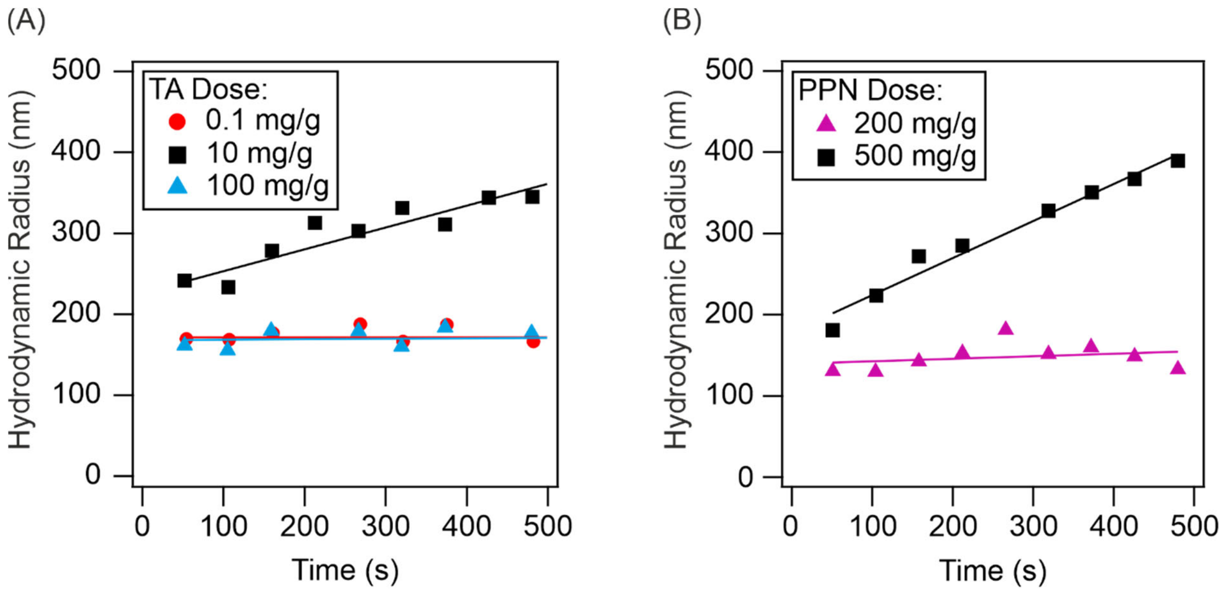

Figure S1. Hydrodynamic radius of LDH versus time at different (A) TA and (B) PPN doses expressed in mg TA or PPN per one gram of particle. The solid lines are linear fits used to calculate the stability ratios values (eq 3). In all experiments, 10 mg/L LDH concentration was used and 1 mM NaCl was applied as background electrolyte.

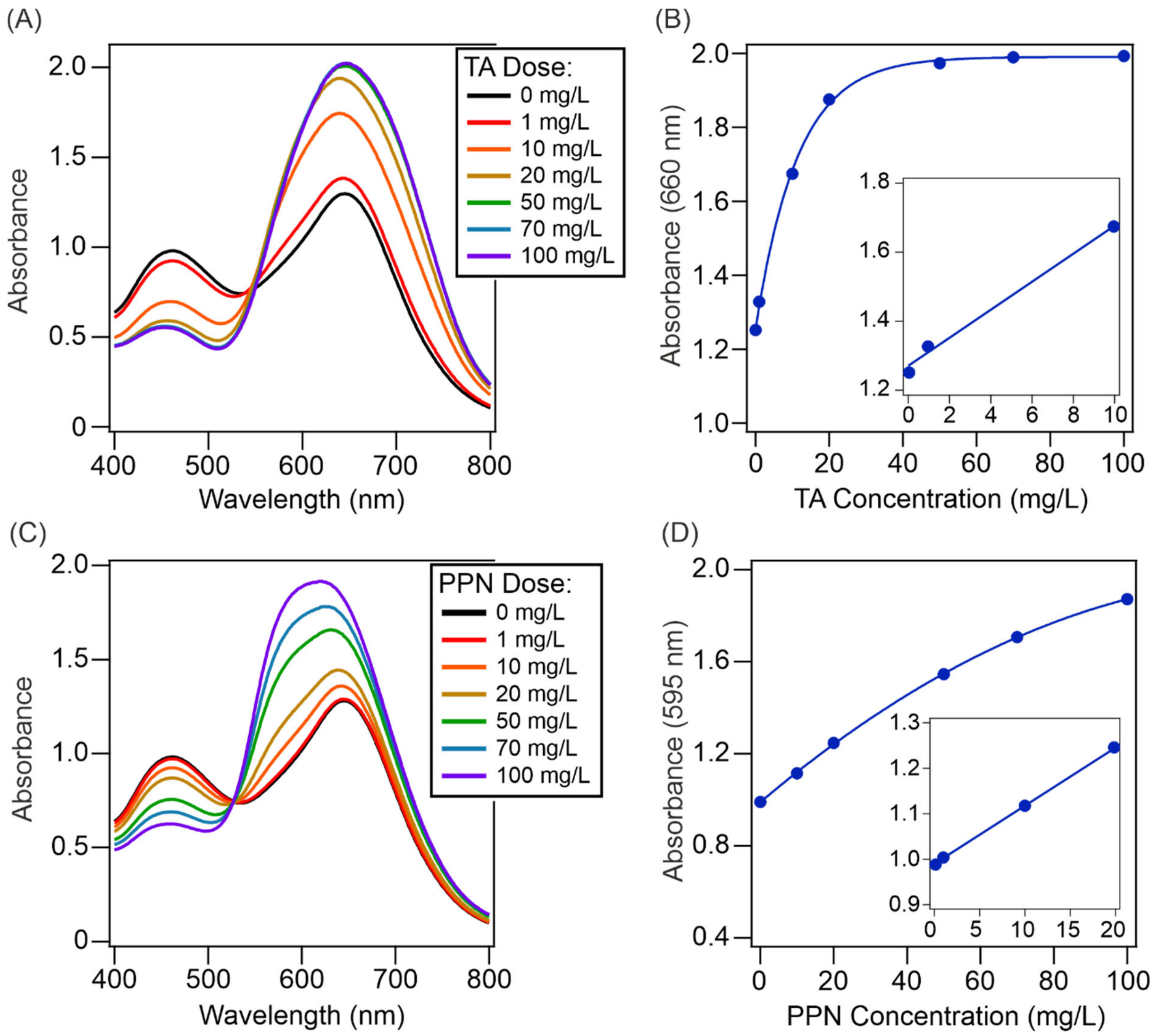

Figure S2. (A) Absorbance spectra of Coomassie Brilliant Blue dye at various TA concentrations. (B) Calibration curve for TA obtained from the absorbance values recorded at 660 nm. The inset represents the linear region. (C) Absorbance spectra of Coomassie Brilliant Blue dye at different PPN concentrations. (D) Calibration curve for TA obtained from the absorbance values recorded at 595 nm. The graph in the inset is the linear section of the data.

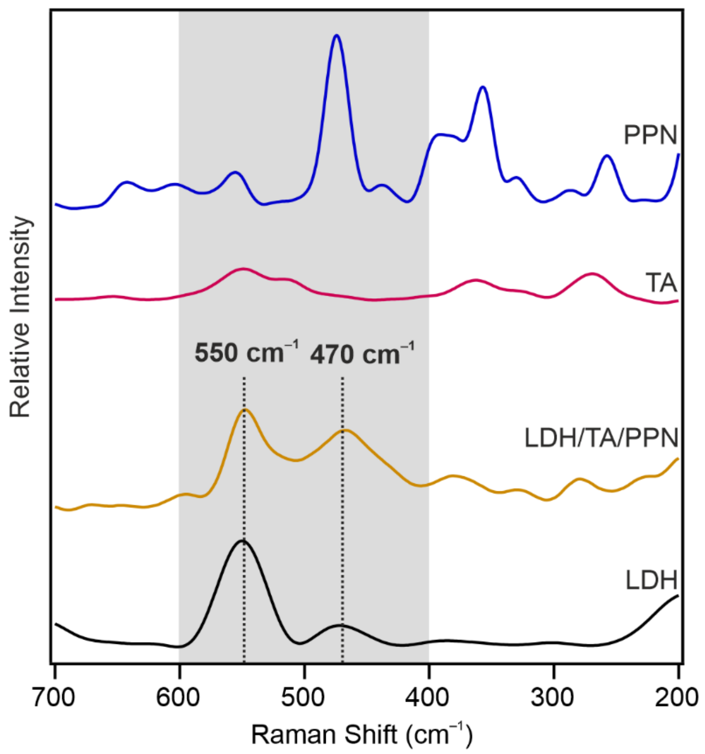

Figure S3. Raman spectra of LDH/LDH/TA/PPN, TA and PPN in the 200-700 cm^{-1} regime.

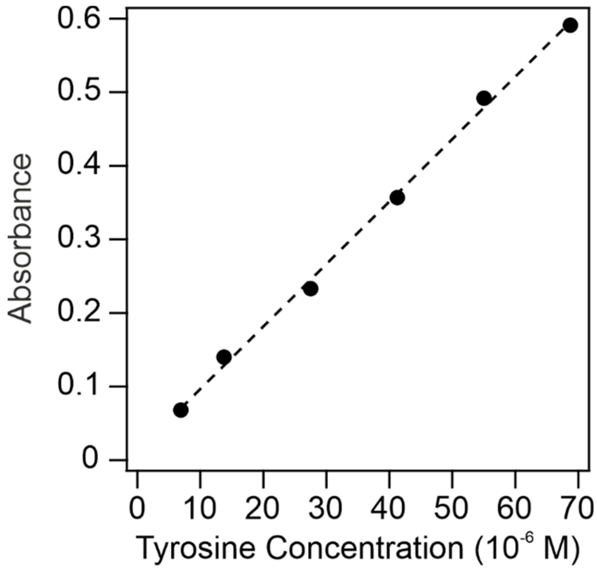

Figure S4. Calibration curve for tyrosine using the protease assay. The absorbance values were recorded at 660 nm. The dashed line is the linear fit used to calculate the concentration data.

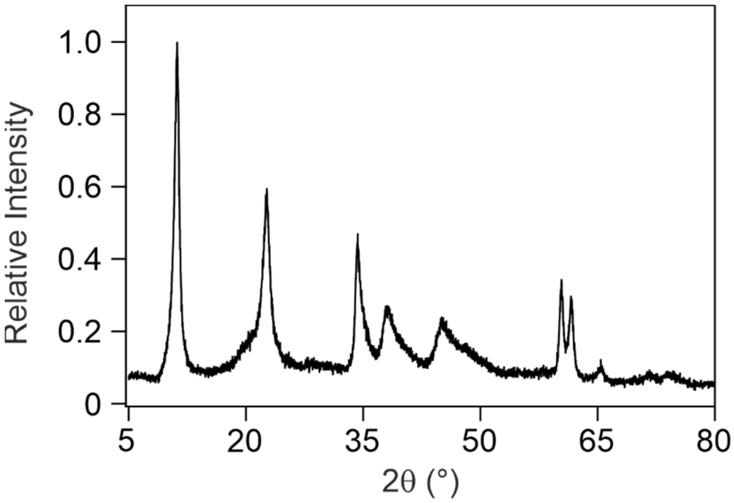

Figure S5. PXRD diffractogram of Mg/Al LDH. The characteristic Miller indices are assigned to the peaks.

Table S1. Assignments of Raman bands determined for LDH, TA, PPN and LDH/TA/PPN nanocomposites.

**Raman Shift (cm^{-1})**	**Assignment**	**Sample**
1048	$\nu(NO_3^-)$	LDH
1039		LDH/TA/PPN
1086	ν(CN)	PPN
1197, 1255	ν(C–C), ν(C–O), ν(C–OH)	TA
1263, 1334	Amide III	PPN
1244, 1345		LDH/TA/PPN
1324, 1370	δ(OH)	TA
1369, 1416, 1442, 1466	ν(CH)	PPN
1612	ν(C=C)	TA
1591		LDH/TA/PPN
1707	ν(C=O)	TA
1680		LDH/TA/PPN
1663	Amide I	PPN
1419, 1499	$\nu(COO^-)$	LDH/TA/PPN

Table S2. Calculated EC_{50} values for TA containing samples at 25°C and 50°C.

Sample	EC_{50} (μM)	
	25˚C	50˚C
TA	0.87 ± 0.08	0.53 ± 0.06
TA-PPN	0.62 ± 0.09	0.50 ± 0.08
LDH/TA/PPN	1.41 ± 0.17	1.12 ± 0.24
